# The MFF-SIRT1/3 axis, regulated by miR-340-5p, restores mitochondrial homeostasis of hypoxia-induced pulmonary artery smooth muscle cells

**DOI:** 10.1038/s41374-022-00730-w

**Published:** 2022-01-18

**Authors:** Chun-Xia Huang, Zhi-Xin Jiang, Da-Yong Du, Zhi-Min Zhang, Yang Liu, Yun-Tian Li

**Affiliations:** 1 The Second School of Clinical Medicine, Southern Medical University, Guangzhou, Guangdong Province PR China; 2 Department of Cardiology, 305 Hospital of PLA, Beijing, PR China; 3 Linfen Peoples’ Hospital, Shanxi Medical University, Linfen, Shanxi Province PR China

**Keywords:** Diseases, Cardiovascular diseases

## Abstract

Mitochondrial dynamics and quality control play a central role in the maintenance of the proliferation–apoptosis balance, which is closely related to the progression of pulmonary arterial hypertension (PAH). However, the exact mechanism of this balance remains unknown. Pulmonary artery smooth muscle cells (PASMCs) were cultured in hypoxia condition for constructing a PAH model in vitro. The expression of genes and proteins were determined by qRT-PCR and western bolt assays. Cell proliferation–apoptosis balance were tested by MTT, EdU and TUNEL assays. The mitochondrial functions were assessed by flow cytometry, JC-1, Mito tracker red staining, and corresponding kits. Besides, the molecular interaction was validated by dual-luciferase reporter assay. MFF was overexpressed in hypoxia-treated PAMSCs. Knockdown of MFF significantly repressed the excessive proliferation but enhanced cell apoptosis in hypoxia-treated PAMSCs. Moreover, MFF silencing improved mitochondrial function of hypoxia-treated PAMSCs by increasing ATP production and decreasing ROS release and mitochondrial fission. Mechanistically, MFF was a directly target of miR-340-5p, and could negatively regulate SIRT1/3 expression. Subsequently, functional rescue assays showed that the biological effects of MFF in hypoxia-treated PAMSCs were negatively regulated by miR-340-5p and depended on the regulation on SIRT1/3 pathway. These results provided evidences that miR-340-5p regulated MFF-SIRT1/3 axis to improve mitochondrial homeostasis and proliferation–apoptosis imbalance of hypoxia-treated PAMSCs, which provided a theoretical basis for the prevention and treatment of PAH.

## Abstract (web summary)

The imbalance between the proliferation and apoptosis of pulmonary arterial smooth muscle cells (PASMCs) is an important pathological process in pulmonary arterial hypertension (PAH). Mitochondrial dynamics and quality control play important roles in maintaining the cell proliferation–apoptosis balance. This paper reveals that miR-340-5p upregulates SIRT1/3 by targeting MFF, therefore improving mitochondrial dysfunction to maintain the proliferation–apoptosis balance of hypoxia-treated PAMSCs.

## Introduction

Pulmonary arterial hypertension (PAH) is a type of pulmonary vascular remodeling disease which characterized by increased pulmonary artery pressure and pulmonary vascular resistance, eventually leading to heart failure and death^1^. Growing evidences have indicated that the imbalance between the proliferation and apoptosis of PAMSCs is a key factor in the development of PAH^2,3^. In eukaryotic cells, mitochondrion is a highly dynamic organelle that participates in lipid biosynthesis, regulating calcium homeostasis, responding to various physiological inputs and genetic stress, and regulating cell proliferation and death^4^. A lot of evidences have revealed that mitochondrial dysfunction is the key cause to result in the imbalance of PASMCs proliferation and apoptosis in PAH^5,6^. Whereas, there are few of mechanism reports have been studied.

Mitochondrial fission factor (MFF) is an important mitochondrial fission regulator, which can interact with dynamin-related protein 1 (Drp1) to mediate the mitochondrial fission process^7^. A previous study has demonstrated that MFF is markedly increased in high glucose-treated cerebrovascular smooth muscle cells (CVSMCs), accompanied by disturbance of mitochondrial dynamics (increased fission and decreased fusion) and CVSMC dysfunction, accelerating cerebral vascular damage^8^. Additionally, Zhang et al. revealed that upregulated MFF in the high-salt rats contributed to mitochondrial dysfunction, and subsequent contractile dysfunction and myocardial apoptosis^9^. However, it remains unclear whether MFF participates in the regulation of PASMCs proliferation and apoptosis.

microRNAs (miRNAs) refer to a class of endogenous noncoding single-stranded RNA molecules with a length of ~22 nt^10^. Increasing evidences have displayed that miRNAs are involved in the development of PAH^11,12^. For example, miR-126 was downregulated in patients with right ventricular failure, and overexpression of miR-126 obviously improved cardiac vascular density and function of PAH animals^13^. Ou et al. showed that miR-340-5p was lowly expressed and its overexpression suppressed interleukin (IL)-1β and IL-6 to alleviate acute pulmonary embolism (APE)-induced PAH^14^. Importantly, Yu et al. study suggested that miR-340 might be a regulator of mitochondria that could inhibit breast cancer cell motility via targeting mitochondrial calcium uniporter^15^. By bioinformatics software prediction, a binding site of miR-340-5p was found in the 3′- untranslated region (UTR) of MFF, which suggested that miR-340-5p might play the role as the upstream regulator of MFF.

Previous work has revealed that MFF silencing effectively preserved mitochondrial homeostasis via amelioration of mitochondrial mitosis by restoration of Sirtuin (SIRT)1/3 expression^16^. SIRT1/3 are nicotinamide adenosine dinucleotide (NAD^+^)-dependent deacetylases located in the cytoplasm and mitochondria, and participated in the processes of vascular remodeling and mitochondrial dysfunction of PAH^17,18^. In the present study, we constructed a PAH model in vitro by hypoxia stimulation PAMSCs, and demonstrated that miR-340-5p negatively regulated MFF to alleviate mitochondrial dysfunction and proliferation–apoptosis balance of hypoxia-stimulated PAMSCs by suppressing SIRT1/3 signaling.

## Materials and methods

### Cell culture

Primary human pulmonary artery smooth muscle cells (PASMCs) were acquired from BeNa Culture Collection (Beijing, China) and were cultured in Dulbecco’s modified eagle’s medium (DMEM; Gibco, MD, USA) containing 10% fetal bovine serum (FBS; Gibco) and 100 U/mL penicillin, and 100 mg/mL streptomycin (Sangon, Shanghai, China) in a humidified atmosphere of 5% CO_2_ at 37 °C. To establish a PAH model in vitro, PASMCs were starved at DMEM medium without serum for 24 h, and then cells were incubated in hypoxic condition of 92% N_2_, 5% CO_2_, and 3% O_2_ for 48 h.

### Cell transfections

The MFF-overexpressing vectors (pcDNA3.1-MFF), small interfering RNA (siRNA) targeting to MFF, SIRT1 and SIRT3 (si-MFF, si-SIRT1, si-SIRT3), miR-340-5p mimics, and miR-340-5p inhibitor as well as their corresponding negative controls (pcDNA3.1-NC, si-NC, inhibitor NC, mimics NC) were synthesized and purchased from Genepharma (Shanghai, China). PAMSCs were maintained in a 96-well plate and the transfection experiments were performed by using Lipofectamine™ 3000 (Invitrogen, CA, USA). After 48 h, the transfection efficiency was measured using qRT-PCR method.

### Quantitative real-time polymerase chain reaction (qRT-PCR)

Total RNA was isolated from cells by using TRIzol reagent (Invitrogen, CA, USA). cDNA was synthesized with HiFiScript cDNA synthesis kit (Toyobo, Osaka, Japan) in accordance with manufacturer’s instruction. Then, the cDNA was used for qRT-PCR assay conducted on an Eppendorf MasterCycler RealPlex4 (Eppendorf, Wesseling-Berzdorf, Germany) using an Ultra SYBR Mixture kit (Toyobo). The relative expression of miRNA and mRNA were respectively normalized by U6 and GAPDH, respectively, and followed by calculated by 2^−ΔΔCT^ method. The primers used in the study were listed as follows (5′–3′):

MFF (F): CACCACCTCGTGTACTTACGC

MFF (R): CCGCTCTCTTTTTAGTCTGCC

SIRT1 (F): TAGCCTTGTCAGATAAGGAAGGA

SIRT1 (R): ACAGCTTCACAGTCAACTTTGT

SIRT3 (F): ACCCAGTGGCATTCCAGAC

SIRT3 (R): GGCTTGGGGTTGTGAAAGAAG

miR-340-5p (F): GCGGTTATAAAGCAATGAGA

miR-340-5p (R): GTGCGTGTCGTGGAGTCG

U6 (F): CTCGCTTCGGCAGCACA

U6 (R): AACGCTTCACGAATTTGCGT

GAPDH (F): TGACCTCAACTACATGGTCTACA

GAPDH (R): CTTCCCATTCTCGGCCTTG

### 3-(4, 5-Dimethylthiazolyl2)-2, 5-diphenyltetrazolium bromide (MTT) assay

Cells were seeded into 96-well plates and cultured in complete DMEM medium for overnight. Then, 5 mg/mL MTT (Sigma-Aldrich, MO, USA) were added and incubated for 4 h at 37 °C. After incubation, the MTT formazan product was dissolved in DMSO (Sigma-Aldrich) and the absorbance at 490 nm was detected by a microplate reader (Bioteke, Beijing, China). The absorbance value relative to the control was taken as the relative cell viability.

### Ethynyl-2ʹ-deoxyuridine (EdU) assay

Cells were plated in 48-well plates at a density of 1.0 × 10^5^ cells per well and cultured for 24 h. Cells were then incubated with EdU (100 μL/well, Sangon) for 2 h. Cells were subsequently fixed with 4% formaldehyde for 25 min, permeabilized with 0.5% Triton X-100 for 25 min and incubated with the Apollo staining solution (100 μL/well) for 30 min protected from light. Hoechst 33342 was employed to indicate the cells by staining their DNA. Cells were observed using a fluorescence microscope (Nikon, Tokyo, Japan).

### Mito Tracker Red staining

Mitochondrial network structure was evaluated using Mito Tracker Red staining (Invitrogen) as described previously^19^. In brief, cells were incubated with 100 nM Mito Tracker Red solution in DMEM for 30 min at 37 °C. Images were obtained using a laser scanning confocal microscope (Olympus Corporation, Tokyo, Japan). The mitochondrial perimeter was measured by the Image J.

### ROS detection

The ROS production in PASMCs was determined by using fluorescent dye mito-SOX (Thermo Fischer Scientific, CA, USA). Cells were stained with 5 μM MitoSOX™ reagent working solution and incubated for 10 min at 37 °C away from light. After treatment, cells were washed three times with warm buffer. Samples were immediately analyzed using flow cytometry.

### Mitochondrial membrane potential detection

5, 5′, 6, 6′-tetrachloro-1, 1′, 3, 3′-tetraethylbenzimidazolcarbocyanine iodide (JC-1) mitochondrial membrane potential fluorescent probe (Yeasen, Shanghai, China) was used to analyze the mitochondrial membrane potential of PASMCs. Cells were plated on a six-well plate at a density of 1 × 10^6^ cells/well. After treatment, the medium was removed, and then cells were incubated with 0.5 mL of JC-1 working solution for 10 min. Then photos were taken using fluorescence inverted microscope (Olympus Corporation). JC-1 monomer (green fluorescence) and aggregate (red fluorescence) form was excited/emitted at a wavelength of 485/530 nm and 485/590 nm, respectively.

### ATP content and caspase-3 activity assays

The ATP level and caspase-3 activity were determined using ATP assay kit (MAK190, Sigma-Aldrich) and caspase activity detection kit (APPA015, Sigma-Aldrich), respectively. All operations were strictly according to the manuals.

### TUNEL staining assay

Cells were fixed by 4% paraformaldehyde, and the apoptotic assay was performed using a TUNEL detection kit (Yeasen, Shanghai, China) according to instructions. After washing by PBS, cells were then treated with DAPI staining solution in accordance with the instruction. After 10 min, DAPI solution was removed and cells were washed by PBS. Cells were observed and photographed under a microscope (Olympus Corporation).

### Western blot assay

The proteins were isolated from cells by using RIPA mixed with 1% protease inhibitor and phosphorylase inhibitor, and concentrations of protein were determined by a BCA Kit (Beyotime, Shanghai, China). Lysate samples were separated using SDS-PAGE, and then transferred to a PVDF membrane. Then, membrane was incubated with primary antibodies including SIRT1 (1: 1000, ab189494, abcam, Cambridge, UK), SIRT3 (1: 1000, ab217319, abcam), and MFF (1:1000, ab129075, abcam). Anti-β-actin antibody (1:5000, ab8226, abcam) was served as a loading control. After washing with PBS-T, the membrane was following incubated with the corresponding secondary antibody labeled with HRP (1:10,000, ab7090, abcam) for 60 min. At last, the membrane was covered with ECL reagents (Beyotime, Shanghai, China) and the images were performed by GEL imaging system (Bio-Rad, CA, USA). The quantification of protein was analyzed by the software Image J.

### Dual-luciferase reporter gene assay

Wild-type (wt) and mutant-type (mut) reporter sequences of MFF 3′-UTR sequences were cloned into PGL3 vector (Genepharma, Shanghai, China). Then, cells were plated onto 24-well plate and were co-transfected with MFF-wt or MFF-mut plasmids and miR-340-5p mimics or mimics NC (Genepharma) by using Lipofectamine™ 3000 (Invitrogen, CA, USA). After transfection for 48 h, the relative luciferase activity was examined using a dual-luciferase reporter assay system (Promega, WI, USA).

### Data analysis

All data was obtained from at least three replicate experiments. Results were expressed as mean ± standard deviation (SD). Statistical analysis was performed using SPSS 19.0 (IBM, NY, USA). The differences among two groups were analyzed by Student’s *t* tests. One-way analysis of variance followed by Turkey’s post test was employed to evaluate the differences among multiple groups. The *p* values <0.05 were considered significant.

## Results

### The establishment and identification of PAH model in vitro

**Fig. 1:**
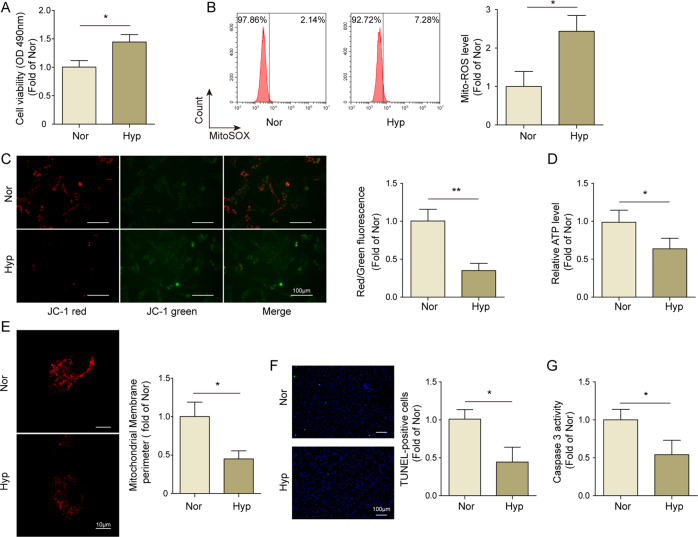
The establishment and identification of PAH model in vitro. **A** MTT assay was performed to determine cell viability of PAMSCs. **B** The level of ROS of PAMSCs was detected using flow cytometry. **C** The mitochondrial membrane potential was determined using JC-1 assay. **D** The content of ATP in PAMSCs was detected using kit. **E** Mito Tracker Red staining was carried out to evaluate the mitochondrial network structure, and the mitochondrial perimeter was measured. **F** TUNEL staining was employed to detect the apoptosis rate of PAMSCs. **G** The activity of caspase 3 in PAMSCs was assessed using kit. The data were expressed as mean ± SD. *n* = 3. *P < 0.05; **P < 0.01, ***P < 0.001.

A hypoxia-induced PAH model in vitro was established as previously reports^3^, then the cell viability, apoptosis and mitochondrial functions were assessed. As shown in Fig. 1A, cell viability of PAMSCs exposed in hypoxia were markedly increased compared to control group. It was also observed that cell proliferation of PAMSCs was remarkably elevated by hypoxic-treatment (Fig. S1A). Subsequently, we also identified that the mitochondrial ROS level was significantly increased while the mitochondrial membrane potential and ATP generation were markedly decreased in hypoxia-stimulated PAMSCs (Fig. 1B–D). Mito Tracker Red staining data revealed that hypoxic-stimulation led to decreased mitochondrial perimeter in PAMSCs, suggesting an increase of mitochondrial fission (Fig. 1E). In addition, apoptotic assay result demonstrated that hypoxic-treatment significantly decreased the TUNLE-positive PASMCs and reduced the activity of caspase 3 (Fig. 1F, G). All the above results suggested that hypoxia-treatment successfully induced mitochondrial dysfunction and proliferation–apoptosis imbalance of PAMSCs in vitro.

### MFF inhibition improved mitochondrial dysfunction and proliferation–apoptosis imbalance in hypoxia-stimulated PAMSCs

**Fig. 2:**
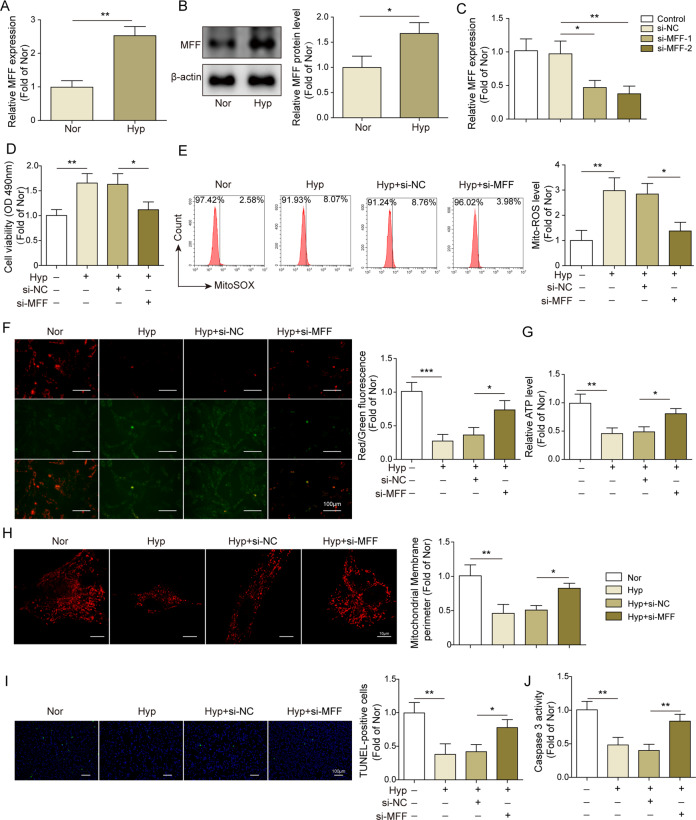
MFF inhibition improved mitochondrial dysfunction and proliferation–apoptosis imbalance in hypoxia-stimulated PAMSCs. **A**, **B** MFF expression in hypoxia-treated PAMSCs was determined using qRT-PCR (**A**) and western blot (**B**) assays, respectively. **C** PAMSCs were transfected with si-NC, si-MFF#1, or si-MFF#2, and then MFF expression was assessed using qRT-PCR assay. PAMSCs were transfected with si-NC or si-MFF, and exposed in hypoxia condition. **D** MTT assay was employed to determine cell viability of PAMSCs. **E** The level of ROS in PAMSCs was detected using flow cytometry. **F** JC-1 assay was performed to evaluate the mitochondrial membrane potential. **G** The content of ATP in PAMSCs was detected using kit. **H** The mitochondrial network structure in PAMSCs was examined by Mito Tracker Red staining, and the mitochondrial perimeter was determined. **I** Cell apoptosis of PAMSCs was evaluated using TUNEL staining. **J** The activity of caspase 3 in PAMSCs was assessed using kit. The data were expressed as mean ± SD. *n* = 3. *P < 0.05; **P < 0.01; ***P < 0.001.

MFF is a mitochondrial fission factor, and its abnormal expression is capable of mediating abnormal mitochondrial division and mitochondrial dysfunction^20^. Here, we observed that both mRNA and protein levels of MFF were markedly increased in hypoxia-treated PAMSCs compared to that in Nor group (Fig. 2A, B). In order to further explore the roles of MFF in the regulation of mitochondrial function, proliferation and apoptosis in vitro, MFF was silenced in hypoxia-treated PAMSCs by transfection of si-MFFs. As showed in Fig. 2C, qRT-PCR result demonstrated that both si-MFF#1 and si-MFF#2 successfully decreased the mRNA level of MFF. Considering si-MFF#2 had a better interference effect, we selected si-MFF#2 (hereinafter collectively referred to as si-MFF) for following experiments. Results from MTT and EdU assays showed that MFF silencing dramatically reduced the excessive cell viability and proliferation of hypoxia-treated PAMSCs (Figs. 2D,  S1B). Similar trends were observed in mitochondrial function detections. Knockdown of MFF significantly decreased ROS release, and increased mitochondrial membrane potential and content of ATP compared with Hyp + si-NC group (Fig. 2E–G). Additionally, MFF knockdown resulted in the increased mitochondrial perimeter but reduced mitochondrial fission and in hypoxia-treated PAMSCs (Fig. 2H). Moreover, apoptotic assays disclosed that knockdown of MFF significantly enhanced TUNEL-positive cell rate and caspase-3 activity of hypoxia-treated PAMSCs (Fig. 2I, J). Taken together, MFF silencing could improve mitochondrial dysfunction and proliferation–apoptosis imbalance in hypoxia-treated PAMSCs.

### The regulatory effects of MFF inhibition in hypoxia-treated PAMSCs partly depended on SIRT1/3 signaling

**Fig. 3:**
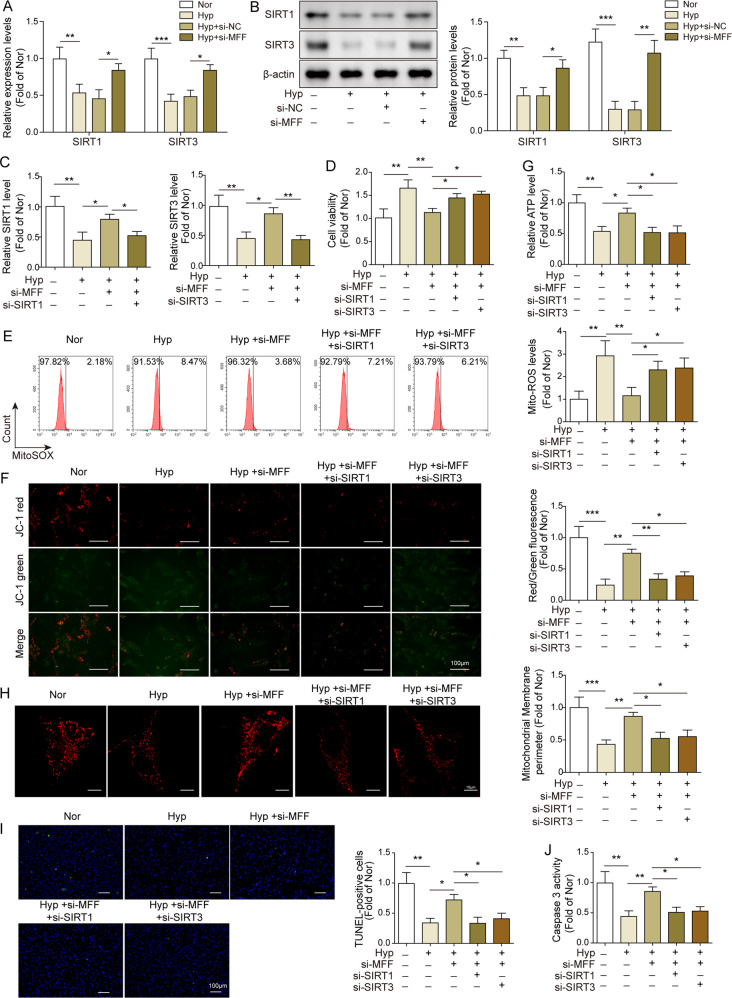
The regulatory effects of MFF inhibition in hypoxia-treated PAMSCs partly depended on SIRT1/3 signaling. **A**, **B** The mRNA and protein expression of SIRT1/3 in hypoxia-treated PAMSCs with or without MFF silence were determined using qRT-PCR and western blot, respectively. **C** The knockdown efficiency of SIRT1 and SIRT3 in MFF silenced hypoxia-treated PAMSCs were assessed using qRT-PCR assay. **D** Cell viability of PAMSCs was evaluated using MTT assay. **E** The level of ROS in PAMSCs was assessed using flow cytometry. **F** The mitochondrial membrane potential was evaluated using JC-1 assay. **G** The content of ATP in PAMSCs was detected using kit. **H** Mito Tracker Red staining was carried out to evaluate the mitochondrial network structure, and the mitochondrial perimeter was measured. **I** Cell apoptosis of PAMSCs was evaluated using TUNEL staining. **J** The caspase-3 activity in PAMSCs was assessed using kit. The data were expressed as mean ± SD. *n* = 3. *P < 0.05; **P < 0.01; ***P < 0.001.

A previous study has shown that MFF inhibition can restore SIRT1/3 expression^16^. Therefore, we first examined the expression of SIRT1/3 in MFF silenced hypoxia-treated PAMSCs. As shown in Fig. 3A, B, we observed that SIRT1 and SIRT3 were obviously decreased in hypoxia-treatment PAMSCs, while their expression were partly restored in MFF silenced hypoxia-treated PAMSCs. In order to further explore the functional relationship between MFF and SIRT1/3 in hypoxia-treated PAMSCs, we both silenced MFF and SIRT1/SIRT3 in hypoxia-treated PAMSCs. As shown in Fig. 3C, the promoting effects on SIRT1/3 caused by si-MFF in hypoxia-treated PAMSCs were obviously eliminated by knockdown of SIRT1/3. Subsequently, proliferative assays showed that silencing of SIRT1/3 significantly reversed the inhibitory effects on cell viability and proliferation mediated by MFF knockdown in hypoxia-treated PAMSCs (Figs. 3D, S1C). Compared to Hyp + si-MFF group, the protective effects of MFF silence on mitochondria functions were eliminated by SIRT1/3 co-silencing, presenting as the increase of ROS release and the decrease of mitochondrial membrane potential and ATP generation (Fig. 3E–G). Besides, SIRT1/3 inhibition remarkably eliminated the inhibitory effects of MFF knockdown on mitochondrial fission and the increase of mitochondrial perimeter (Fig. 3H). Moreover, SIRT1/3 inhibition dramatically reversed the promotion effects of si-MFF on cell apoptosis and caspase-3 activity of hypoxia-treated PAMSCs (Fig. 3I, J). In summary, these finding demonstrated that SIRT1/3 signaling involved in the regulatory network of MFF in PAH progression.

### MFF-SIRT1/3 axis was regulated by miR-340-5p

**Fig. 4:**
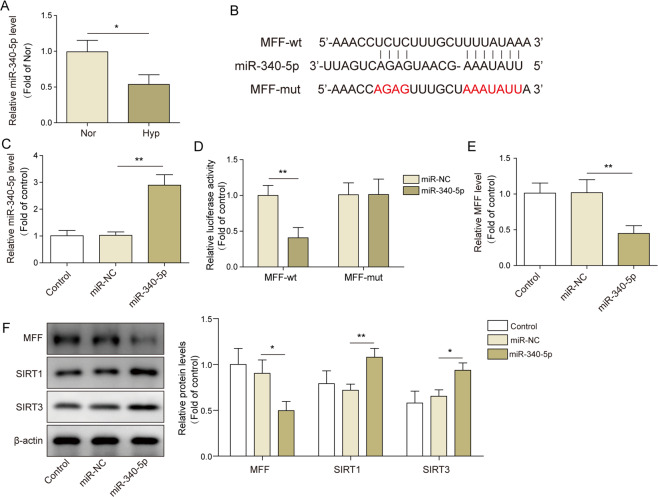
MFF-SIRT1/3 axis was regulated by miR-34-5p. **A** The expression of miR-340-5p in hypoxia-treated PAMSCs was determined using qRT-PCR. **B** Targetscan software was applied to predict the binding site between miR-340-5p and MFF. **C** The transfection of miR-340-5p mimics was measured by qRT-PCR assay. **D** PAMSCs were cultured and co-transfected with MFF-wt or MFF-mut plasmids and miR-340-5p mimics or mimics NC, then cell luciferase activity was measured. **E** The mRNA level of MFF in miR-340-5p overexpressed PAMSCs was detected by qRT-PCR. **F** The protein levels of MFF, SIRT1 and SIRT3 were detected by western blot. The data were expressed as mean ± SD. *n* = 3. *P < 0.05; **P < 0.01; ***P < 0.001.

To elucidate whether miRNAs is participated in the regulation of MFF expression, we first examined the expression of miR-340-5p in hypoxia-induced PAMSCs after bioinformatics prediction. As shown in Fig. 4A, the expression of miR-340-5p was markedly decreased in hypoxia-treated PAMSCs. Subsequently, starbase online database (http://starbase.sysu.edu.cn/) predicted the binding site between miR-340-5p and MFF 3′-UTR (Fig. 4B). Next, qRT-PCR assay result showed that miR-340-5p expression was markedly enhanced after transfection with miR-340-5p mimics (Fig. 4C). Dual-luciferase reporter gene assay displayed that luciferase activity in MFF-wt group was obviously suppressed following treated with miR-340-5p mimics, while the luciferase activity in the MFF-mut group remained no significant difference (Fig. 4D). Furthermore, both mRNA and protein level of MFF were significantly downregulated after miR-340-5p overexpression, while the protein levels of SIRT1 and SIRT3 were elevated (Fig. 4E, F). In conclusion, these data evidenced that miR-340-5p could negatively regulate MFF and activate SIRT1/3 expression.

### miR-340-5p protected mitochondrial function and proliferation–apoptosis balance in hypoxia-treated PAMSCs via downregulating MFF

**Fig. 5:**
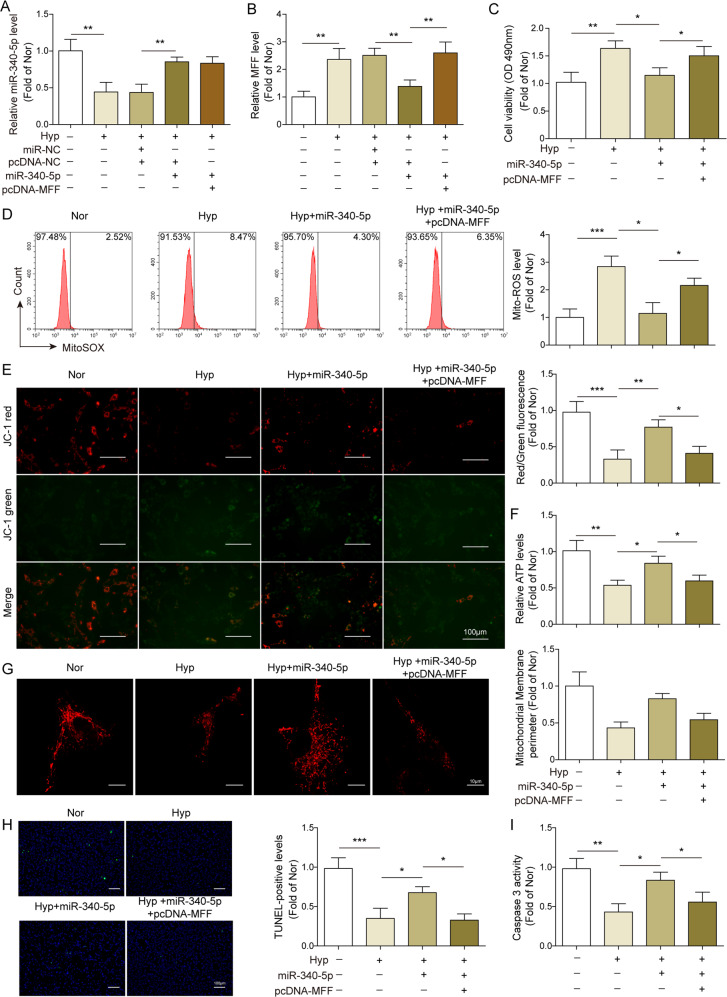
miR-340-5p protected mitochondrial function and proliferation–apoptosis balance in hypoxia-treated PAMSCs via downregulating MFF. Hypoxia-treated PAMSCs were transfected with miR-340-5p mimics or co-transfected with pcDNA-MFF and miR-340-5p mimics. **A**, **B** The expression of miR-340-5p and MFF in hypoxia-treated PAMSCs were assessed using qRT-PCR. **C** MTT assay was employed to determine cell viability of PAMSCs. **D** The level of ROS in PAMSCs was assessed using flow cytometry. **E** The mitochondrial membrane potential was evaluated using JC-1 assay. **F** The content of ATP in PAMSCs was detected using kit. **G** The mitochondrial network structure in PAMSCs was examined by Mito Tracker Red staining, and the mitochondrial perimeter was determined. **H** Cell apoptosis of PAMSCs was evaluated using TUNEL staining. **I** The caspase-3 activity in PAMSCs was assessed using kit. The data were expressed as mean ± SD. *n* = 3. *P < 0.05; **P < 0.01; ***P < 0.001.

Next, we wanted to explore the effects of MFF on miR-340-5p-mediated biological functions in vitro. Therefore, we co-overexpressed miR-340-5p and MFF in hypoxia-treated PAMSCs. qRT-PCR results displayed that miR-340-5p mimics transfection dramatically increased miR-340-5p expression in hypoxia-induced PAMSCs, and overexpression of MFF markedly abolished the inhibitory effect of miR-340-5p on MFF expression (Fig. 5A, B). Subsequently, functional experiments discovered that miR-340-5p overexpression markedly decreased the excessive viability and proliferation of hypoxia-treated PAMSCs, while the effects were significantly reversed by co-overexpression of MFF (Figs. 5C, S1D). Mitochondrial functions assay also presented that miR-340-5p overexpression reduced ROS release, elevated mitochondrial membrane potential and content of ATP of hypoxia-treated PAMSCs by downregulating of MFF level (Fig. 5D–F). In addition, Mito Tracker Red staining result revealed that hypoxia-treatment resulted in increased mitochondrial fission and decreased mitochondrial perimeter in PAMSCs, while these effects were abolished by miR-340-5p upregulation, however, the effect mediated by miR-340-5p upregulation was greatly weakened by MFF co-overexpression (Fig. 5G). Similarly, the elevated TUNEL-positive cell rate and caspase-3 activity were observed in miR-340-5p overexpressed hypoxia-treated PAMSCs, while MFF overexpression obviously reduced these effects (Fig. 5H, I). In summary, miR-340-5p exerted a protective role in mitochondrial functions and proliferation–apoptosis balance of hypoxia-treated PAMSCs by inhibiting MFF.

## Discussion

Mitochondrial dysfunction is mainly measured by mitochondrial fusion-division imbalance, reduced energy production, and excessive ROS production^21^. Recently, growing evidence has displayed that mitochondrial dysfunction is closely related to the occurrence and development of many human diseases, including PAH^22–25^. For example, Culley and Chan study revealed that mitochondria in pulmonary vascular cells in PAH exhibited decreased oxidative phosphorylation and increased glycolysis, consistent with the Warburg effect^26^. This mitochondrial dysfunction in PAH resulted in extensive remodeling, increased cell proliferation, and decreased cell apoptosis, which further causing increased pulmonary vascular resistance and pulmonary artery pressures^26–28^. Therefore, exploring the regulatory mechanism of mitochondrial dysfunction in PAH is of great significance to the discovery of PAH therapeutic targets. In the present study, we further confirmed that hypoxia resulted in increased ROS production, decreased mitochondrial membrane potential, and decreased ATP synthesis in PAMSCs, indicating that the PAH model in vitro was successfully established.

MFF an important type of Drp1 adaptor protein, is a key component of the mitochondrial division mechanism^29^. Otera et al. revealed that MFF was essential for mitochondrial recruitment of Drp1 during mitochondrial fission in mammalian cells^7^. Specifically, MFF overexpression enhanced mitochondrial fission via mitochondrial recruitment of Drp1^7^. However, the roles of MFF in PAH have not been fully studied. In our study, we found that MFF expression was dramatically increased in hypoxia-treated PAMSCs, which agreed with previous study^29^. Additionally, our results suggested that inhibition of MFF decreased mtROS production, increased ATP generation and mitochondrial membrane potential of hypoxia-treated PAMSCs, thus ameliorating proliferation–apoptosis imbalance in hypoxia-treated PAMSCs. All these observations suggested that MFF might be a potential intervention target for PAH.

SIRT family is a family of NAD^+^ dependent deacetylases in mammals, composed of SIRT1-SIRT7^30^. Among them, SIRT1 and SIRT3 are the most important deacetylases in the cytoplasm and mitochondria, respectively^18,31^. It has reported that SIRT1/3 could deacetylate mitochondria-related multiple enzymes and electron transport chain complexes, thus participating in the regulation of mitochondrial biological processes such as energy metabolism, oxidative stress and mitochondrial dynamics^18,32–34^. For instance, Singh et al. demonstrated that SIRT1 and SIRT3 could eliminate ROS by regulating the main antioxidant enzymes such as SOD, CAT and GPX, thereby reducing oxidative damage^35^. Tian et al. also showed that SIRT1 could activate PGC-1α to increase ATP content and SOD enzyme activity and reduce ROS and MDA level, thus improving mitochondrial energy metabolism and oxidative stress of cardiomyocytes^36^. In addition, the activation of SIRT1 and SIRT3 could activate the ATP production and the mitochondrial antioxidant function in skeletal muscle^37^. Furthermore, it was reported that SIRT1/SIRT3 deficiency resulted in impaired mitochondrial fusion and decreased mitochondrial perimeter in cardiomyocyte^38^. More importantly, as previously reported, the deficiency of SIRT1/3 have identified to promote the progression of PAH^17,18^. For instance, Paulin et al.’s study revealed that the depletion of SIRT3 increased acetylation and inhibition of mitochondrial enzymes and complexes, which suppressed mitochondrial function and led to the PAH development^17^. Consistently, SIRT1^−/−^ mouse showed a more intense vascular remodeling after hypoxia exposure, which was associated with an increase in right ventricle pressure and hypertrophy^18^. In the present study, we presented that SIRT1/3 were obviously downregulated in hypoxia-treated PAMSCs. Interestingly, similar to Wang et al.’s study^16^, we found that SIRT1/3 were negatively regulated by MFF, and participated in the regulation of mitochondrial functions and proliferation–apoptosis balance of hypoxia-treated PAMSCs mediated by MFF, which suggesting that SIRT1/3 might be a downstream signaling of MFF in PAH progression.

MiRNAs are reported to mainly regulate the expression of target genes at the post-transcriptional level to participate in the processes of PAH^39,40^. For example, Caruso et al. demonstrated that miR-124 enhanced endothelial cell glycolysis in PAH via targeting polypyrimidine tract binding protein 1 (PTBP1) and pyruvate kinase M2 (PKM2)^39^. By bioinformatic prediction, miR-340-5p was validated as an upstream regulator of MFF/SIRT1/3 axis. Moreover, it was reported that miR-340-5p was lowly expressed in plasma of PAH patients, and its overexpression could suppress PAH via targeting IL-1β and IL-6^14^. Consistent with the previous study, our results proved that miR-340-5p overexpression improved mitochondrial dysfunction and proliferation–apoptosis imbalance in hypoxia-PAMSCs, which was abolished by MFF overexpression. These finding implied miR-340-5p might play as a protective factor in PAH.

In conclusion, our research provided the evidences to support that MFF silence regulated by miR-340-5p restored mitochondrial homeostasis and proliferation–apoptosis balance in hypoxia-induced PAMSCs through activating SIRT1/3 signaling, which might provide some theoretical basis for MFF. Thus, MFF might serve as a potential therapeutic target for clinical prevention and treatment of PAH.

## Supplementary information

Figure S1

## Data Availability

All data generated or analyzed during this study are included in this article. The datasets used and/or analyzed during the current study are available from the corresponding author on reasonable request.
