# Supplementary figures and images for: The MFF-SIRT1/3 axis, regulated by miR-340-5p, restores mitochondrial homeostasis of hypoxia-induced pulmonary artery smooth muscle cells
Source: Lab Invest. 2022 Jan 18;102(5):515–23. doi: 10.1038/s41374-022-00730-w (PMC9042702; doi:10.1038/s41374-022-00730-w)

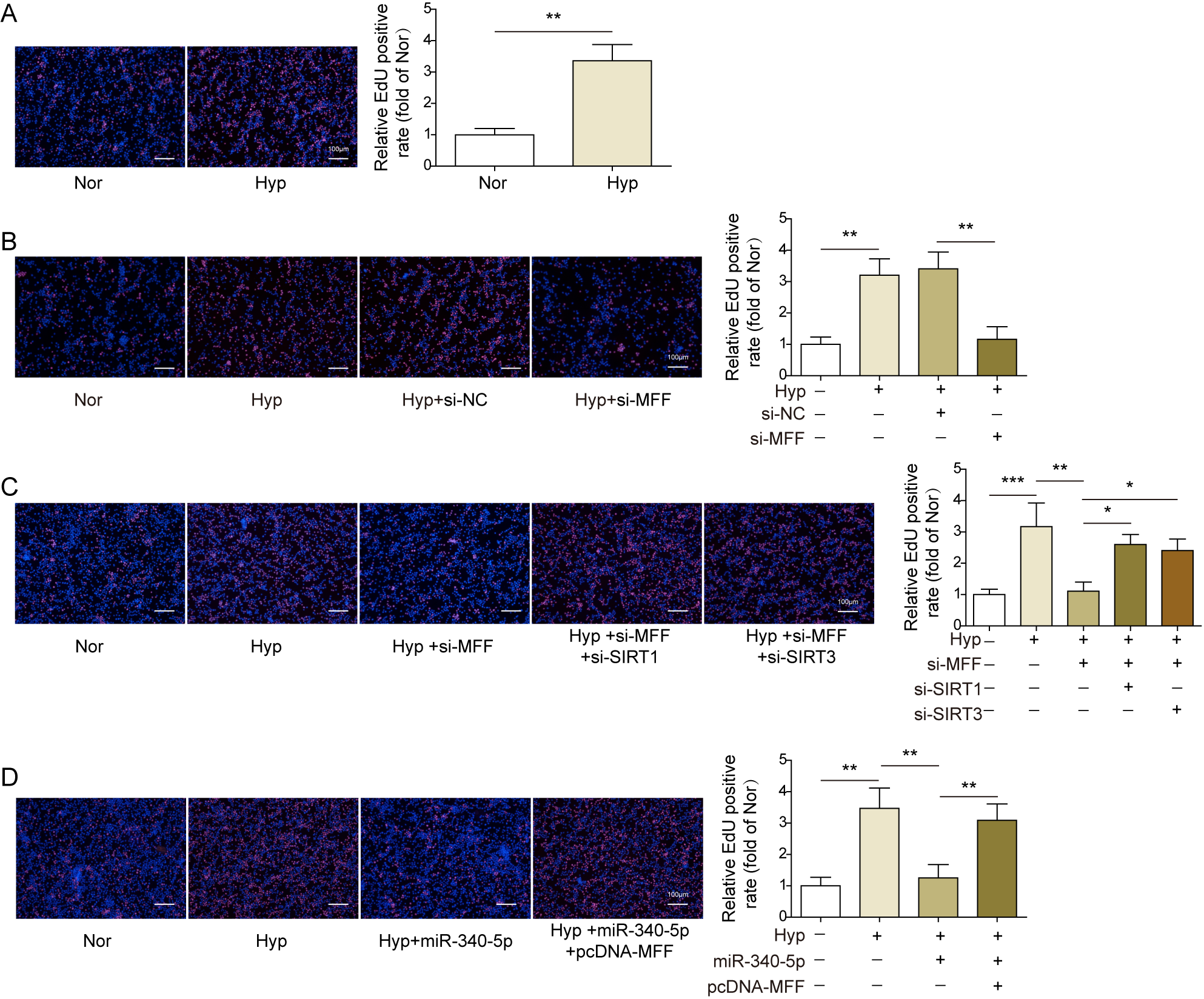

Supplement: Supplementary file 1 — Figure S1 [file 41374_2022_730_MOESM1_ESM.tif]
